# Cyanidin‐3‐Glucoside Supplementation Modulates Bone Resorption in Experimental Apical Periodontitis

**DOI:** 10.1096/fba.2025-00199

**Published:** 2025-10-13

**Authors:** Rafaela Ricci, Romulo de Oliveira Sales‐Junior, Maria Antônia Leonardo Pereira Neta, Bharbara de Moura Pereira, Julissa Denisse Arguello Alvarado, Nathália Evelyn da Silva Machado, Luciano Tavares Ângelo Cintra, Edilson Ervolino, Anil Kishen, João Eduardo Gomes‐Filho

**Affiliations:** ^1^ Department of Preventive and Restorative Dentistry School of Dentistry, São Paulo State University (UNESP) Araçatuba São Paulo Brazil; ^2^ Department of Basic Science School of Dentistry, São Paulo State University (UNESP) Araçatuba São Paulo Brazil; ^3^ Department of Dentistry, Faculty of Dentistry University of Toronto Toronto Ontario Canada

**Keywords:** apical periodontitis, bone resorption, cyanidin‐3‐glucoside, inflammation, polyphenols

## Abstract

Apical periodontitis (AP) is an inflammatory/infectious disease that leads to alveolar bone resorption. Cyanidin‐3‐glucoside (C3G) is an anthocyanin known for its anti‐inflammatory, antioxidant, and bone metabolism‐regulating properties, suggesting benefits during AP development. This study aimed to evaluate the influence of C3G supplementation on bone resorption and inflammation during the progression of AP in Wistar rats. Sixteen male albino Wistar rats were divided into two groups: a control group (C) that received potable water, and a treatment group (C3G) that received a C3G solution. Supplements were administered daily via oral gavage for 45 days. AP was induced in both groups on Day 15, and it was allowed to develop for 30 days. After the experimental period, the animals were euthanized, and the upper and lower jaws were collected to assess lesion volume, bone microstructure, the intensity of the inflammatory infiltrate, and the expression of IL‐1β, TNF‐α, IL‐10, the RANKL/OPG ratio, and TRAP immunolabeling at the AP site. Statistical analysis was performed with a 5% significance level. Compared to controls, C3G‐treated rats exhibited reduced lesion volume, increased bone volume fraction and trabecular thickness, lower RANKL/OPG ratio, and fewer TRAP‐positive cells/mm (*p* < 0.05). No significant differences were observed in inflammatory infiltrate or cytokine expression (IL‐1β, TNF‐α, and IL‐10) at the lesion site (*p* > 0.05). C3G supplementation modulated bone resorption by reducing lesion volume and osteoclast activity, while enhancing bone microstructural parameters, without significantly affecting local inflammatory markers.

## Graphical abstract

Cyanidin‐3‐glucoside (C3G) supplementation was administered to Wistar rats for 45 days. Apical periodontitis (AP) was induced on Day 15 and allowed to progress for 30 days. Compared with controls, C3G‐treated animals showed a lower RANKL/OPG ratio and fewer TRAP‐positive cells, along with improved bone parameters, including increased bone volume fraction (BV/TV) and trabecular thickness (Tb.Th). These changes were associated with reduced lesion volume, demonstrating that C3G effectively modulated osteoclast activity and bone resorption during AP development.

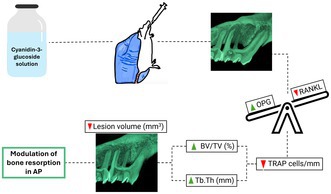

## 1 Introduction

Apical periodontitis (AP) is an inflammatory condition that often progresses asymptomatically and affects nearly half of the adult population, frequently presenting with multiple lesions [1]. It begins in response to bacterial infection of the dental pulp, leading to tissue necrosis and activation of the host immune response in the periapical region. As the infection progresses, bacterial components stimulate immune cells to release proinflammatory cytokines, which promote osteoclastic activity, accelerate bone resorption, and hinder reparative bone formation [2].

Bone remodeling is primarily regulated by the coordinated activity of osteoblasts, which are responsible for bone formation, and osteoclasts, multinucleated cells involved in bone resorption and typically identified by tartrate‐resistant acid phosphatase (TRAP) staining [3]. Osteoblasts modulate osteoclastogenesis through the production of receptor activator of nuclear factor kappa‐B ligand (RANKL) and osteoprotegerin (OPG) as its decoy receptor. RANKL is crucial for osteoclast differentiation, activation, and survival. In contrast, OPG binds to RANKL, preventing its interaction with RANK on osteoclast precursors and thereby inhibiting bone resorption. A balance in the RANKL/OPG ratio is essential for proper bone homeostasis [4].

Cyanidin‐3‐glucoside (C3G) is an anthocyanin—a subclass of polyphenols found in fruits, vegetables, and red wine—that has been associated with bone‐protective effects. These include reducing bone loss, promoting osteoblast proliferation, enhancing mineralization and osteocalcin expression, and increasing ERK phosphorylation [5, 6, 7]. In addition, C3G exhibits antioxidant and anti‐inflammatory properties in various disease models [8, 9, 10, 11, 12, 13].

Previous studies have shown that the systemic administration of some polyphenol combinations or complexes can reduce inflammation and bone resorption at periapical lesion sites [14, 15, 16]. Recent studies have also highlighted the use of local immune‐modulatory agents to enhance healing after treatment of AP. However, the effects of C3G on AP have not been explored. Therefore, this study aimed to evaluate the influence of C3G supplementation on bone resorption and inflammation during the development of AP in Wistar rats. The null hypothesis was that the systemic administration of C3G would not affect bone resorption or inflammation in experimentally induced AP.

## 2 Materials and Methods

### 2.1 Experimental Animals

The study was submitted and approved by the Institutional Animal Care and Use Committee of Araçatuba Dental School (document number 443‐2023). Sixteen male Wistar rats ( *Rattus norvegicus* , Wistar, RRID: RGD_13508588), aged 3 months and weighing 250–300 g each, were included in this study. They were housed in a temperature‐controlled environment (22°C ± 1°C, 70% humidity) with a 12‐h light–dark cycle and fed throughout the entire experimental period with a solid diet and water ad libitum.

The sample size was estimated based on parameters from previous studies [14, 15, 16]. An alpha error of 0.05% and 95% power were used to determine that a minimum of seven animals per group was necessary to recognize a significant difference. To account for potential complications that may arise during the study, we added one more animal to each group. This adjustment resulted in 16 animals that were randomly divided into two groups, totaling eight rats per group. Subject selection criteria included healthy male Wistar rats of a defined age and weight range, as described above. All animal experiment protocols were conducted in compliance with the ARRIVE 2.0 guidelines for reporting in vivo experiments.

### 2.2 Diet Administration

The animals from the C group received potable water as a sham. The animals from the C3G (SigmaAldrich, PHL89616, CAS7084244PHL89616) group received an aqueous solution containing 2.3 mg/L of C3G dissolved in 2 mL of alcohol and mixed with 998 mL of distilled water (the same quantity present in red wine) according to the results of the quantification analysis of phenolic components [15].

The administration was carried out daily in the morning at a dosage of 4.28 mL/kg body weight [14, 15]. For all groups, both waters provided via water dispenser and solid feed (Labina‐Purina, Paulínia, Brazil) remained freely available throughout the experiment. The solutions were administered through oral gavage for 45 days as a supplement to the conventional diet, starting 15 days prior to the induction of AP and continuing for 30 days after the induction of the disease.

### 2.3 Induction of Apical Periodontitis

All animals were anesthetized with ketamine (80 mg/kg, Avenco Inc., Fort Dodge, IA) and xylazine (4 mg/kg, Mobay Corp., Shawnee, KS), and received induction of AP on Day 15. The coronal opening of the first and second upper and lower molars on the right side (four teeth) was carried out using a carbon steel bur (Long Neck Ln Bur—Maillefer, Dentsply) with a diameter of 0.5 mm, ensuring standardization of all pulp exposures to a 0.5 mm diameter. The teeth remained open, and the lesion was analyzed 30 days after the induction, which characterizes a chronic periapical lesion [17, 18].

### 2.4 Sample Collection

At the end of the experimental stage, the animals were again anesthetized following the same previously described protocol and euthanized using an overdose of anesthetic solution [15]; their right‐side upper and lower jaws were removed. Upper jaws were immediately frozen for microtomography analysis, and lower jaws were fixed in 10% neutral buffered formalin for subsequent histological and immunohistochemical processing. No animals were lost or excluded during the experimental period.

### 2.5 Microtomographic Analysis

The upper right jaws were subjected to micro‐computed tomography (Micro‐CT) to evaluate bone resorption associated with AP, using the SkyScan 1272 system (Bruker, Kontich, Belgium). Three‐dimensional (3D) reconstructions were performed using NRecon software (Bruker, Kontich, Belgium) and Data Viewer (version 1.4.4, 64‐bit). Quantitative analysis was performed using CTAnalyser software (version 1.12.4.0, Bruker MicroCT, 2012). The defined volume of interest encompassed the periapical region of the distal root, and the extent of bone resorption was calculated and reported in cubic millimeters (mm^3^) [15].

The bone microstructure in the region of interest was quantitatively assessed through standard 3D morphometric parameters: bone volume fraction (BV/TV), trabecular thickness (Tb.Th), trabecular number (Tb.N), and trabecular separation (Tb.Sp), as previously described by de Oliveira Sales‐Junior et al. [16], in accordance with established guidelines [19]. BV/TV reflects the proportion of mineralized bone within the total tissue volume analyzed, Tb.Th corresponds to the mean thickness of trabecular structures, Tb.N represents the number of trabeculae per unit length, and Tb.Sp indicates the mean distance between trabeculae. Morphometric analyses were performed using CTAn software (SkyScan, Bruker), and 3D reconstructions were generated using CTvox software (SkyScan, version 2.7) in sagittal, coronal, and axial planes to visualize AP lesions.

### 2.6 Histological Analysis of the Inflammatory Infiltrate

The fixed lower right‐side jaws were decalcified in a 10% EDTA solution, embedded in paraffin, and sectioned at a thickness of 5 μm, following a standard histology protocol. Hematoxylin and eosin staining were used for the analysis of the inflammatory infiltrate in the periapical area of the distal root of the lower right first molar. The intensity of the inflammation was analyzed based on the approximate mean number of inflammatory cells in the most representative region of the lesion, as observed at 400× magnification adjacent to the tooth apex. The inflammatory infiltrate was scored as follows: 0 (absent or few inflammatory cells), Score 1 (up to 25 cells and mild reaction), Score 2 (25–125 inflammatory cells and moderate reaction), and Score 3 (more than 125 cells and severe reaction) [20].

### 2.7 Immunohistochemical Analysis

Immunohistochemical analysis was performed using the immunoperoxidase technique as previously described [14]. Interleukin‐1 beta (IL‐1β), tumor necrosis factor‐alpha (TNF‐α), interleukin‐10 (IL‐10), and the RANKL/OPG ratio were analyzed in the periapical lesion of the first molar distal root at 400× magnification. Immunostaining was defined as brown staining in the cytoplasm of cells and the extracellular matrix, and scored as follows: 0—complete absence of immunoreactive (IR) cells; 1—low IR cells and weak extracellular matrix staining; 2—moderate IR cells and moderate extracellular matrix staining; and 3—high number of IR cells and intense extracellular matrix staining. The RANKL/OPG ratio was calculated by dividing the RANKL score by the OPG score for each sample. TRAP‐positive cells were quantified at the perimeter and expressed as cells per millimeter (cells/mm) [20]. Analyses were conducted by a blinded, calibrated investigator (E.E.) using an optical microscope (Optiphot‐2, Nikon).

### 2.8 Statistical Analysis

Statistical analysis was performed using GraphPad Prism 8.0 software (GraphPad Prism, RRID: SCR_002798, San Diego, CA, USA). The normality of distribution for continuous quantitative variables was assessed using the Shapiro–Wilk test. Variables following a normal distribution were expressed as mean ± standard deviation, while those with a non‐normal distribution were presented as median, minimum, and maximum values. For quantitative data with normal distribution, Student's *t*‐test was applied. For histological and immunohistochemical analyses, the Mann–Whitney U test was used. A significance level of 5% (*p* < 0.05) was adopted for all statistical analyses.

## 3 Results

### 3.1 Lesion Volume and Bone Microstructure

**FIGURE 1:**
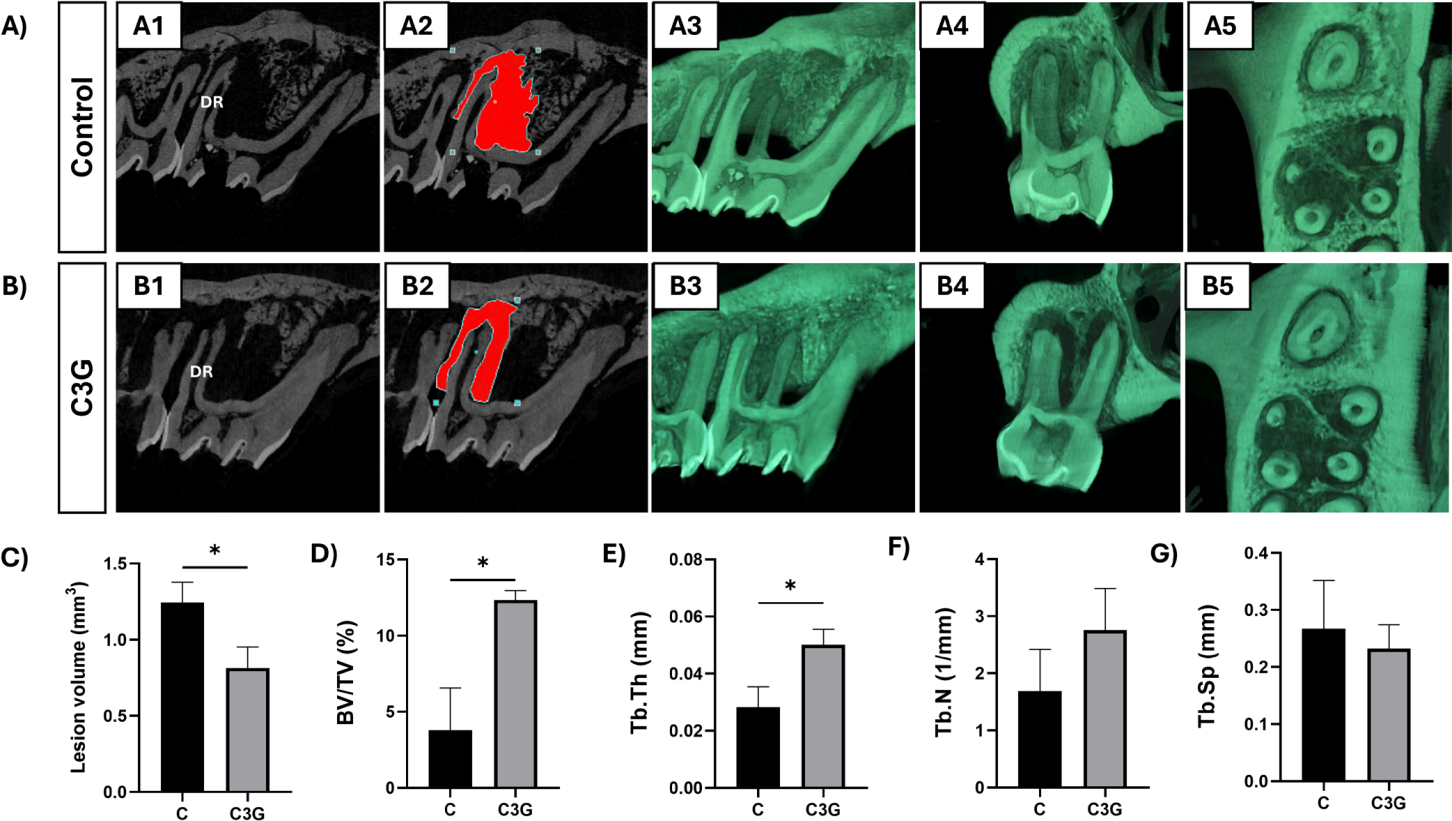
Micro‐CT reconstructions illustrate periapical lesions in the distal root (DR) of the upper first molar in the Control group (A1–A5) and the C3G group (B1–B5). Images A1, A2, B1, and B2 show 2D sagittal slices highlighting the lesion volume (red area) defined for analysis. Reduced bone resorption is observed in the C3G group. Images A3 and B3 show 3D reconstructions in the sagittal plane; A4 and B4 show the coronal plane; and A5 and B5 show the axial plane. Microtomographic data plots (C–G): Bar graphs show the mean and standard deviation for each group regarding lesion volume (C), bone volume fraction (BV/TV) (D), trabecular thickness (Tb.Th) (E), trabecular number (Tb.N) (F), and trabecular separation (Tb.Sp) (G). Statistical differences are indicated by *.

Micro‐CT images and corresponding quantitative data are presented in Figure 1. By Day 30, all animals exhibited increased hypodense periapical areas on Micro‐CT, consistent with AP development. The C3G group showed a significantly smaller mean lesion volume (0.847 ± 0.141 mm^3^) compared to the C group (1.172 ± 0.198 mm^3^) (*p* < 0.0044). Bone volume fraction (BV/TV) and the trabecular thickness (Tb.Th) were significantly higher in the C3G group (BV/TV = 12.346 ± 0.619; Tb.Th = 0.0502 ± 0.005) than in the C group (BV/TV = 3.790 ± 2.774; Tb.Th = 0.0284 ± 0.007) (*p* < 0.05). The trabecular number (Tb.N) followed a similar trend, being higher for the C3G group (2.759 ± 0.730), though not different from the C group (1.691 ± 0.731) (*p* > 0.05). No significant difference was observed in trabecular separation (Tb.Sp) (*p* > 0.05).

### 3.2 Inflammatory Infiltrate

**FIGURE 2:**
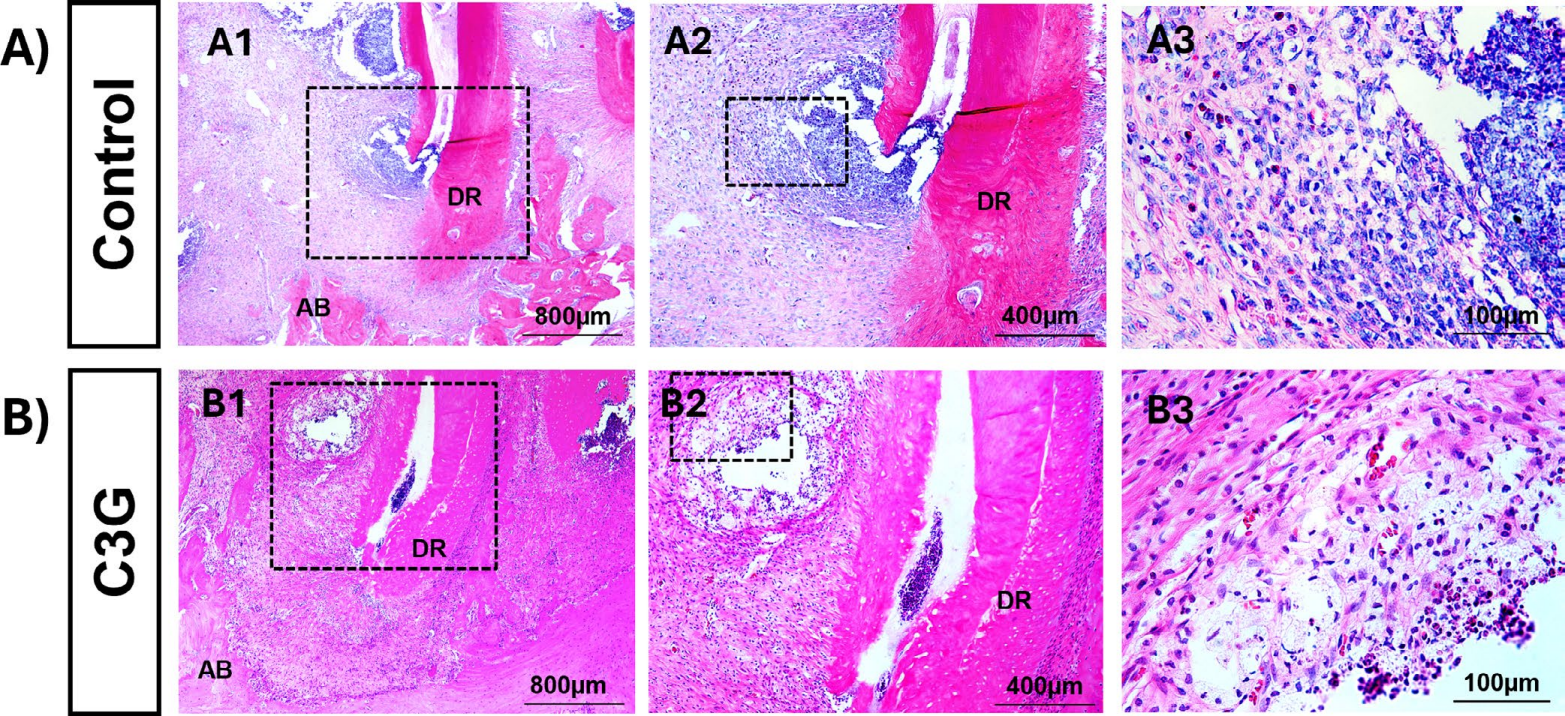
The photomicrographs show the histologic aspects and the alveolar bone (AB) resorption of the apical regions around the foraminal opening of the distal root (DR) of the lower first molar. The severe inflammatory process can be observed for the Control group (A1–A3), and a moderate inflammatory process can be observed for the C3G group (B1–B3). Hematoxylin and eosin staining. Rectangles indicate the enlarged area in the next magnification (50×, 100×, and 400×, respectively). Scale bars: 800 μm (A1 and B1), scale bars: 400 μm (A2 and B2), and scale bars: 100 μm (A3 and B3).

**FIGURE 3:**
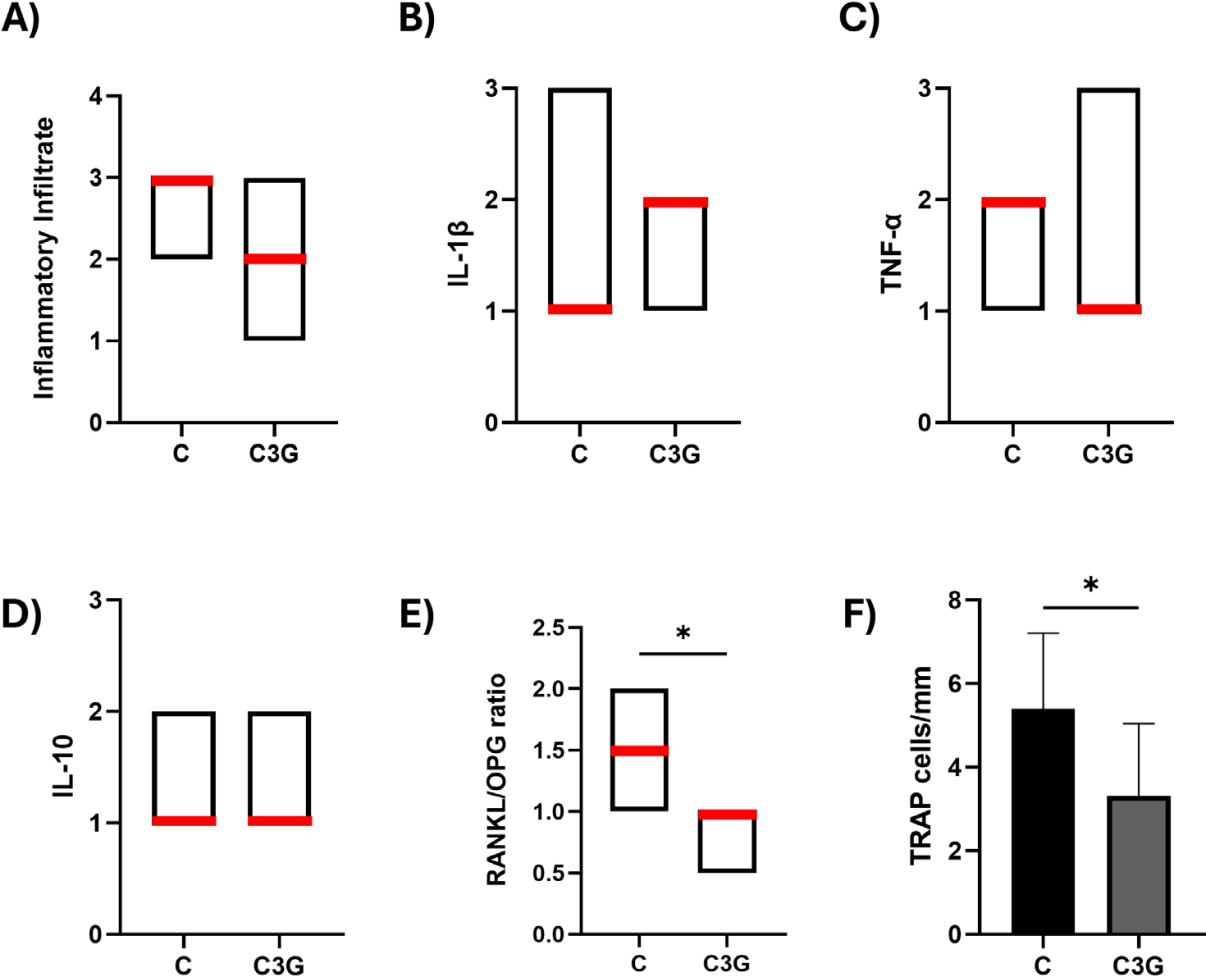
The red line in the charts represents the median of each group for the intensity of the inflammatory infiltrate (A), and for the immunolabeling of IL‐1β (B), TNF‐α (C), IL‐10 (D), and the RANKL/OPG ratio (E). The bar graph shows the mean and standard deviation of each group for the number of TRAP‐positive cells per millimeter (F). Statistical differences are indicated by *.

Figure 2 shows representative histological sections, while Figure 3 presents the median inflammatory scores with their respective ranges. In both the C3G and C groups, inflammatory infiltrates, necrosis, and bone resorption were observed, confirming the development of AP. The median inflammatory score was 3 in the C group and 2 in the C3G group; however, the difference between the groups was not statistically significant (*p* > 0.05).

### 3.3 IL‐1β, TNF‐α, IL‐10, RANKL/OPG Ratio and TRAP Immunostaining

**FIGURE 4:**
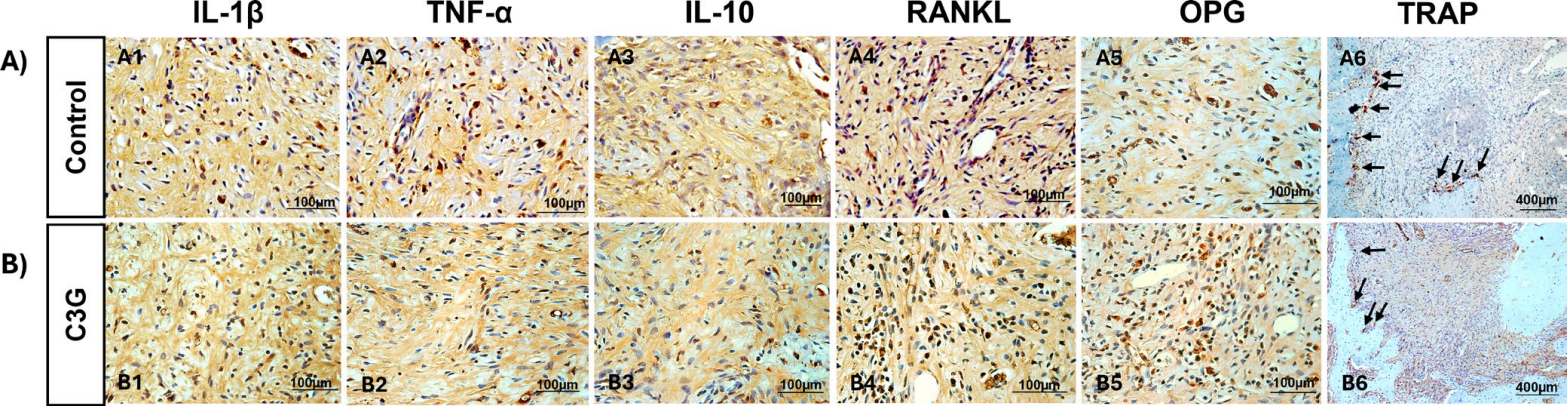
The photomicrographs show immunostaining for IL‐1β (A1, B1), TNF‐α (A2, B2), IL‐10 (A3, B3), RANKL (A4, B4), OPG (A5, B5), and TRAP (A6, B6) in the periapical lesion around the foraminal opening of the distal root of the lower first molar in the Control group (A) and the C3G group (B). Arrows indicate TRAP‐positive cells at the periphery of the periapical lesion (A6, B6). Counterstaining: Harris hematoxylin. Original magnification for IL‐1β, TNF‐α, IL‐10, RANKL, and OPG: 400× (scale bars: 100 μm); for TRAP: 100× (scale bars: 400 μm).

The immunoreactivity patterns for IL‐1β, TNF‐α, IL‐10, RANKL/OPG ratio, and TRAP are summarized below and presented in Figures 3 and 4. For IL‐1β, Score 1 predominated in the C group and was not significantly different from the C3G group, which showed a median score of 2 (*p* > 0.05). TNF‐α showed a median score of 2 in the C group, while the C3G group had a score of 1; however, this difference was not statistically significant (*p* > 0.05). For IL‐10, both groups exhibited a mix of scores 1 and 2, with no significant difference between them (*p* > 0.05). Regarding the RANKL/OPG ratio, the C group demonstrated a significantly higher ratio (median score of 1.5) compared to the C3G group, which had a reduced ratio (median score of 1) (*p* = 0.0326). Finally, TRAP immunolabeling was specific to osteoclasts, and the C group showed a significantly higher number of TRAP‐positive multinucleated cells/mm (5.394 ± 1.813) in the periapical region compared to the C3G group (3.312 ± 1.731) (*p* = 0.0483).

## 4 Discussion

This study evaluated the potential benefits of C3G on bone resorption and inflammation in AP. Given that recent studies have demonstrated protective effects of red wine polyphenols on bone resorption and inflammation associated with AP [14, 15, 16], the specific effects of C3G—a polyphenol known for its antioxidant and anti‐inflammatory properties, as well as its ability to modulate bone metabolism and promote bone formation [5, 6, 7]—were further investigated. Although C3G did not alter the intensity of the inflammatory infiltrate or the immunostaining of inflammatory cytokines in this study, it did modulate bone metabolism at the AP site, thereby rejecting the null hypothesis.

Periapical lesions were induced by pulp exposure, as initially described by Yamasaki et al. [18] and consistently adopted in recent animal studies [14, 15, 16]. This well‐established method simulates pulp contamination and the subsequent development of AP in humans. Thirty days were selected for analysis, as it corresponds to complete pulp necrosis and characterizes a chronic stage of the disease in rats [17, 18].

Micro‐CT analysis confirmed the development of AP in both groups and revealed a reduced lesion volume in C3G‐treated animals compared to controls. Given that larger radiolucencies increase the risk of lesion persistence by 2.3 times within 4 years, smaller lesions may be associated with better treatment outcomes [21, 22]. In addition, C3G supplementation preserved bone microstructure, as evidenced by higher bone volume fraction (BV/TV), trabecular number (Tb.N), and trabecular thickness (Tb.Th) compared to the control group. Similar protective effects on bone microstructure were also observed in rats with induced AP supplemented with either dealcoholized red wine or açaí, both of which are rich sources of C3G [16, 23]. Together, these findings suggest a protective effect of C3G on bone during AP development, contrasting with the typical bone loss observed as the disease progresses [24]. Additionally, C3G supplementation resulted in a reduced RANKL/OPG ratio. This ratio is widely used as an indicator of local osteolytic activity, and its reduction suggests a decrease in stimulation of osteoclast differentiation and bone resorption [4]. This finding is further supported by the lower number of TRAP‐positive cells observed at the periphery of the periapical lesions in rats treated with C3G compared to those receiving water (control).

TRAP is an enzymatic marker used to identify and quantify mature osteoclasts, the primary cells responsible for bone resorption [3]. These results align with the C3G‐mediated inhibition of osteoclastogenesis, potentially through modulation of the RANKL‐MAPK (ERK, JNK, p38) pathway, followed by downregulation of c‐Fos and NFATc1, without affecting the production of osteoclast differentiation factors such as OPG by osteoblasts in vitro [6]. C3G has also been shown to promote bone formation by upregulating osteoblast differentiation marker genes and enhancing bone matrix production in vitro, supporting its role not only as an inhibitor of osteoclastogenesis but also as a positive regulator of osteoblast differentiation [6]. Other studies reinforce these findings, demonstrating that C3G promotes osteoblast proliferation and enhances late‐stage differentiation via ERK1/2 activation, as evidenced by increased osteocalcin expression and mineralization capacity in vitro [7].

Histological analysis confirmed the presence of periapical lesions in all animals after 30 days, characterized by inflammatory infiltrate and bone resorption. In this study, the C3G group showed a median inflammation score of 2, while the C group presented a median score of 3; however, this difference was not statistically significant. Although C3G is known for its anti‐inflammatory effects, such modulation has mainly been reported in other inflammatory models, often involving different routes of administration, phenolic metabolites, or higher polyphenol doses [25]. The dose used in this study was intentionally chosen to reflect the concentrations naturally found in dietary sources, such as red wine, where C3G content has been previously quantified [15]. In contrast, anti‐inflammatory effects have typically been observed at doses ranging from 10 to 200 mg/kg in various tissues and experimental conditions [26, 27, 28, 29]. Similarly, recent findings in rats supplemented with açaí—rich in C3G, its major anthocyanin—showed beneficial effects on alveolar bone, without significant changes in the periapical inflammatory infiltrate observed in histological analysis [23].

The evaluation of the periapical lesion in this study occurred during a chronic inflammatory phase that may not yet have stabilized. This phase is characterized by sustained vascular, cellular, and immunological activity. At this stage, the active inflammatory environment may limit detectable modulatory effects, which have only been observed after a longer period of lesion development [16]. Additionally, significant bone‐resorbing activity has been found in human chronic periapical lesions [30]. This observation helps explain why CG3 has been limited in its ability to significantly modulate inflammatory intensity and the associated markers in periapical lesions. IL‐1β plays a crucial role in bone resorption related to periodontal disease and is typically elevated at actively destructive sites but not in inactive ones [30, 31, 32]. However, IL‐1β‐expressing cells were not detected at any stage of periapical lesion development in rats, indicating that bone resorption can occur independently of inflammatory cell infiltration [33]. While TNF‐α has been identified in chronic lesions, it appears to exist at levels that are insufficient to induce significant bone resorption [30]. In contrast, IL‐10 functions as a regulatory anti‐inflammatory cytokine [34]. Notably, the modulation of IL‐1β, TNF‐α, and IL‐10 by C3G has primarily been demonstrated in vitro or in vivo using serum, biological fluids, or tissue homogenates analyzed through ELISA and qPCR, rather than through immunohistochemistry [26, 27, 35, 36, 37, 38].

Some limitations of this study should be taken into account. First, the dose of C3G administered differed from those typically used in experimental models. Additionally, analyses were conducted 30 days after the induction of AP, which corresponds to a chronic phase characterized by ongoing lesion progression and active inflammation [15, 16, 17]. This timing may have limited the detection of any modulatory effects. Lastly, while rodent models provide valuable mechanistic insights, direct extrapolation of the findings to humans remains challenging.

Despite these limitations, this is the first study to assess the effects of C3G on bone resorption and inflammation in the context of AP. Our findings suggest that C3G supplementation modulates bone metabolism, as evidenced by decreased lesion volume, increased bone volume fraction (BV/TV), greater trabecular thickness (Tb.Th), a lower RANKL/OPG ratio, and fewer TRAP‐positive cells at the lesion periphery. However, no significant changes were observed in the intensity of the inflammatory infiltrate or in the immunolabeling of IL‐1β, TNF‐α, and IL‐10 at the lesion site. Overall, these results imply that the beneficial effects of C3G are primarily mediated through the regulation of osteoclast and osteoblast activity, rather than through direct modulation of local inflammatory mediators.

## Author Contributions

R.R., R.d.O.S.‐J., B.d.M.P., J.D.A.A., N.E.d.S.M., and J.E.G.‐F. conceptualized the study; R.R., R.d.O.S.‐J., M.A.L.P.N., B.d.M.P., J.D.A.A., and N.E.d.S.M. performed the experiments; L.T.Â.C. performed apical periodontitis induction. E.E. contributed to histologic and immunohistochemistry analysis; R.R., A.K., and J.E.G.‐F. analyzed the data and wrote the original draft. All authors reviewed, edited the manuscript, and approved the final version.

## Conflicts of Interest

The authors declare no conflicts of interest.

## Data Availability

The data that support the findings of this study are available from the corresponding author upon reasonable request.
